# Molecular Targets of TRAIL-Sensitizing Agents in Colorectal Cancer

**DOI:** 10.3390/ijms13077886

**Published:** 2012-06-25

**Authors:** Carmine Stolfi, Francesco Pallone, Giovanni Monteleone

**Affiliations:** Department of Systems Medicine, University of “Tor Vergata”, Via Montpellier 1, Rome 00133, Italy; E-Mails: pallone@uniroma2.it (F.P.); gi.monteleone@med.uniroma2.it (G.M.)

**Keywords:** DR4, DR5, caspase-8, p53, CHOP, survivin, extrinsic pathway, intrinsic pathway, chemotherapeutics, natural products

## Abstract

Tumor necrosis factor (TNF)-related apoptosis inducing ligand (TRAIL), a member of the TNF superfamily, interacts with its functional death receptors (DRs) and induces apoptosis in a wide range of cancer cell types. Therefore, TRAIL has been considered as an attractive agent for cancer therapy. However, many cancers are resistant to TRAIL-based therapies mainly due to the reduced expression of DRs and/or up-regulation of TRAIL pathway-related anti-apoptotic proteins. Compounds that revert such defects restore the sensitivity of cancer cells to TRAIL, suggesting that combined therapies could help manage neoplastic patients. In this article, we will focus on the TRAIL-sensitizing effects of natural products and synthetic compounds in colorectal cancer (CRC) cells and discuss the molecular mechanisms by which such agents enhance the response of CRC cells to TRAIL.

## 1. Introduction

Induction of programmed cell death or apoptosis in tumor cells is a pivotal mechanism for most anti-cancer approaches including chemotherapy, radiation or immunotherapy [1]. However, conventional anti-cancer strategies are associated with several adverse effects due to their toxicity also in non-transformed cells [1]. Since its discovery in 1995, tumor necrosis factor (TNF)-related apoptosis inducing ligand (TRAIL/Apo2L), a member of the TNF superfamily [2], has been involved in cancer biology. TRAIL induces apoptosis in malignant cells both *in vitro* [3] and in pre-clinical models of cancer [4,5]. Moreover, mutations in genes of the TRAIL pathway machinery are associated with human tumorigenesis [6–9]. TRAIL shows very little or no toxicity towards normal cells [4]. The reason why normal cells are resistant to TRAIL-induced apoptosis is not yet known, but there is evidence that non-neoplastic cells express high levels of decoy receptors (DcR)s for TRAIL, which could interfere with TRAIL signaling [10]. Nonetheless, cells derived from many human cancers, such as colorectal cancer (CRC), are resistant against TRAIL-driven apoptosis due to defects in the TRAIL signaling machinery (e.g., down-regulation and/or impaired functionality of TRAIL receptors, increased level of anti-apoptotic proteins) [11]. Restoring the susceptibility of CRC cells to TRAIL could thus help improve the ways we manage patients with this neoplasia.

In this article, we discuss the molecular mechanisms by which various natural products and synthetic compounds increase the susceptibility of CRC cells to TRAIL-induced apoptosis.

## 2. TRAIL Signaling Pathway and Mechanisms of TRAIL Resistance

TRAIL is known to bind five different receptors [12]. Two of these receptors, DR4 (also named TRAIL-R1) and DR5 (also named TRAIL-R2 or KILLER), are coined DRs due to the presence of a cytoplasmic death domain which allows triggering of apoptosis upon TRAIL binding [13]. DcR1, also termed TRAIL-R3 or TRID, and DcR2 (TRAIL-R4, TRUNDD) are expressed on the cell surface but lack a functional intracellular death domain. Both receptors confer protection against TRAIL-induced apoptosis rather than delivering apoptotic signals [14] and may be up-regulated by p53 or hypoxia in CRC cells [15,16]. The fifth TRAIL-receptor is osteoprotegerin, a secreted, low affinity receptor for TRAIL, suggested to mediate TRAIL resistance even though its physiological relevance is still a matter of debate [17]. Binding of TRAIL to DR4 and DR5 leads to caspase-8 activation through Fas-associated death domain (FADD) in the death-inducing signaling complex (DISC) [3]. Activated caspase-8 can induce apoptosis through the so-called extrinsic pathway by directly triggering downstream effector caspases (*i.e*., caspase-3, -6 and -7) [18]. DRs can also indirectly activate effector caspases through the intrinsic mitochondria-mediated apoptotic pathway [19]. In this later case, activated caspase-8 cleaves Bid thereby generating truncated Bid (tBid). tBid in turn translocates to the mitochondria where it interacts with Bax and Bak, thereby promoting the release of pro-apoptotic factors, such as cytochrome c and Smac/DIABLO [19]. Cytochrome c together with Apaf-1 and caspase-9 form a functional apoptosome that results in cleavage and activation of caspase 9 [20]. Active caspase-9 cleaves and activates executioner caspases (e.g., caspases-3) leading to apoptosis [19]. In parallel, Smac/DIABLO can bind to and dampen the activity of cellular inhibitor of apoptosis protein (IAP) members [21] which are powerful caspase inhibitors.

**Figure 1:**
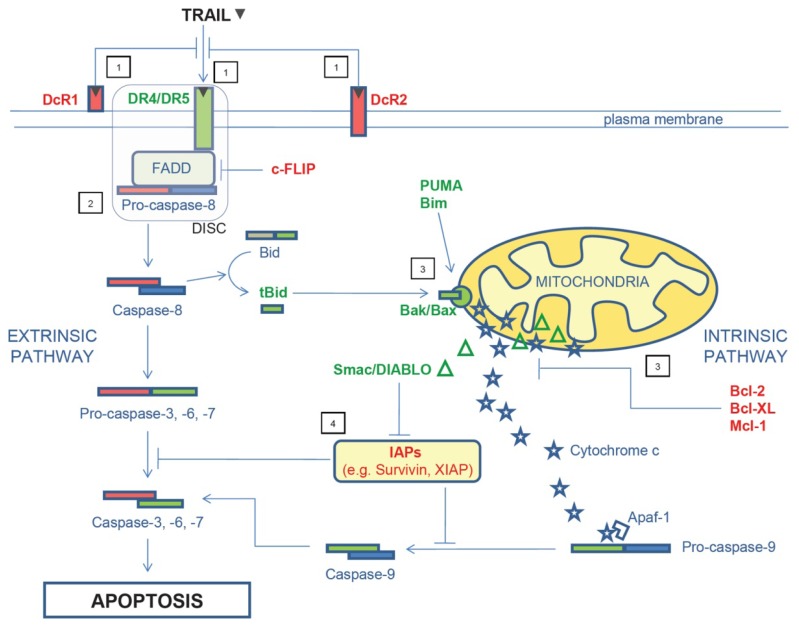
TRAIL signaling pathway and mechanisms of TRAIL resistance. Binding of TRAIL to DR4 or DR5 leads to activation of caspase-8 through FADD. Activated caspase-8 can directly activate effector caspase-3, -6, and -7 via the extrinsic pathway or cleave Bid and activate the intrinsic mitochondria-mediated pathway. Initiation of the latter pathway leads to activation of effector caspases through caspase-9. Activation of caspase-3, -6, and -7 leads to apoptosis. TRAIL resistance can occur at several levels. At the outer membrane, TRAIL sensitivity can be reduced by DcR1 and/or DcR2 over-expression as well as by defective expression/function of DR4 and/or DR5 (1). At the DISC, down-regulation, degradation or inactivation of the initiator caspase-8 and/or increased expression of c-FLIP can lead to TRAIL resistance (2). At the mitochondria, defects in pro-apoptotic molecules (e.g., Bid, Bax, Bak, PUMA, Bim) and/or up-regulation of proteins able to prevent cytochrome c and Smac/DIABLO release (e.g., Bcl-2, Bcl-xL and Mcl-1) also interfere with TRAIL signaling (3). Finally, members of the IAP family prevent the activation of both caspase-9 and caspase-3, -6 and -7 (4). →indicates activation, ⊥ indicates inhibition.

Many cancer cells are resistant to apoptotic signals triggered by TRAIL. The mechanism underlying TRAIL resistance is not fully understood, but it is supposed to rely on defects occurring at various levels in the TRAIL signaling pathway. The first mechanism involves DR expression/function. For example, malignant cells may have diminished expression of DR4 and DR5 due to defective p53 given that p53 directly regulates the transcription of both DRs through specific p53 binding sites [22]. Down-regulation of DR surface expression could be also due to impaired transport from ribosomes [23]. Plasma membrane microdomains enriched in cholesterol and glycosphingolipids (*i.e*., lipid rafts) play an important role in clustering or aggregating surface DRs into membrane complexes at specific sites and in the initiation of DR–induced apoptosis [24,25]. Therefore defective redistribution/relocalization of DR4 and DR5 in lipid rafts on the outer membrane could contribute further to the weak response of cells to TRAIL [26]. Finally, impaired DR functionality due either to loss-of-function mutations or epigenetic changes has been documented in neoplastic cells [27]. A second mechanism relates to defects in the expression/activity of molecules which control DISC function. These include down-regulation, degradation or inactivation of the initiator caspase-8 [28] as well as perturbations of the physiological level of cellular FLICE-like inhibitory protein (c-FLIP), a molecule that is co-recruited with caspase-8 to the DISC and inhibits caspase-8 release-dependent pro-apoptotic signals [29,30]. A low ratio caspase-8/c-FLIP has been described in many CRC cell lines and coupled with increased resistance to TRAIL-induced apoptosis [31]. Further defects involve intrinsic pathway-related pro-apoptotic proteins (e.g., Bim, Bid, Bax, Bak, PUMA) and molecules able to prevent mitochondria outer-membrane permeabilization and cytochrome c release, such as the anti-apoptotic Bcl-2 family members [e.g., Bcl-2, Bcl-xL and myeloid cell leukemia sequence 1 (Mcl-1)] [31]. Finally, resistance of cancer cells to TRAIL could be secondary to enhanced expression of IAPs. Indeed, members of the IAP family [e.g., X-linked IAP (XIAP), cellular IAP1 (c-IAP1), c-IAP2, survivin] prevent activation of both caspase-3 and caspase-9 thus inhibiting at the same time both the extrinsic and intrinsic apoptotic pathway [32]. IAPs, and particularly survivin and XIAP, are up-regulated in CRC [33,34] and thought to represent a major hurdle for TRAIL-based therapies in this neoplasia. Figure 1 summarizes the TRAIL signaling pathway and the above-mentioned mechanisms of TRAIL resistance.

## 3. TRAIL-Sensitizing Agents in CRC

In recent years numerous chemotherapeutics, natural products and newly synthesized molecules have been screened for their ability to restore TRAIL sensitivity in cancer cells with encouraging results. Table 1 summarizes the agents reported to overcome TRAIL resistance and synergize with TRAIL in inducing CRC cell death.

### 3.1. Chemotherapeutics–DNA Damage Agents

Chemotherapeutic drugs are widely used in clinical oncology and have been extensively studied as TRAIL sensitizing agents. Enhanced TRAIL DISC formation by several chemotherapeutics allows restoration of TRAIL-induced apoptosis in CRC cells. In particular, cisplatin, VP16 and 5-fluorouracil (5-FU) sensitize HCT-116 and SW480 cells to TRAIL mainly by increasing caspase-8 recruitment and activation at the DISC [39]. Enhanced FADD and pro-caspase-8 recruitment to the DISC as well as activation of the mitochondria-dependent death pathway underlie the TRAIL-sensitizing effect of cisplatin, 5-FU and doxorubicin in HT-29 cells [35,36]. Among the above-mentioned cytotoxic drugs, only 5-FU induces c-FLIP degradation both in HCT-116 [37,39] and HT-29 cells [36] thus contributing to TRAIL sensitization. Additionally, a recent paper indicated that 5-FU sensitizes HCT-116 cells to TRAIL through the induction of DR4 [38]. Knock-down experiments showed that 5-FU requires both p53-dependent and -independent mechanisms to efficiently trigger apoptosis as well as a functional FADD/caspase-8/Bid axis [38]. Otherwise, the specific cyclo-oxygenase-2 inhibitor DuP-697 overcomes TRAIL resistance in HT-29 cells by inducing DR5 clustering at the cell surface and the redistribution of the DISC components (*i.e*., DR5, FADD, and pro-caspase-8) in peculiar lipid rafts called caveolae [25]. TRAIL also synergizes with irinotecan (CPT-11), an analog of the DNA topoisomerase I inhibitor camptothecin, in inducing apoptosis in HCT-116 cells [40]. This later event associates with DR4 and DR5 up-regulation and enhanced degradation of p21, a negative regulator of the apoptotic process. Semisynthetic retinoid fenretinide promotes DR5 up-regulation through the endoplasmic reticulum (ER) stress-related CAAT/enhancer-binding protein homologous protein (CHOP) thus sensitizing CRC cells to TRAIL-driven apoptosis [41]. More recently, lapatinib, a dual inhibitor of EGFR and HER2, was reported to increase TRAIL-induced cell death in CRC cells by promoting JNK/c-Jun-driven DR over-expression [42]. Modulation of molecules involved in the intrinsic/mitochondrial pathway accounts for the sensitization of TRAIL resistant CRC cells to sunitinib, a tyrosine kinase inhibitor, and oxaliplatin, a third-generation platinum agent [43,44]. In particular, sunitinib decreases the expression of the anti-apoptotic proteins Mcl-1 and XIAP [43] while the oxaliplatin-mediated TRAIL sensitization increases phosphorylation of Bcl-xL, thus reducing the anti-apoptotic activity of this protein [44]. However, it is noteworthy to underline that CRC cells harboring functional p53 (e.g., HCT-116) fail to trigger apoptosis upon oxaliplatin and TRAIL treatments due to a strong p53-dependent, oxaliplatin-mediated, up-regulation of DcR1 [75].

### 3.2. Natural Products

Since chemotherapeutic drugs lead to DNA damage in cells, combination treatment of TRAIL with these cytotoxic agents, even at lower doses than those used in conventional chemotherapy, can result in severe side effects [76]. Therefore in the last decade, numerous researchers have focused attention on natural products, which exhibit TRAIL sensitizing effects at concentrations easy to obtain and which appear to be safe [77].

#### 3.2.1. Polyphenols and Related Compounds

Among natural products, polyphenols, and in particular the subgroup of flavonoids, constitute the major category of molecules used in combination with TRAIL in CRC cells. Food polyphenols sensitize CRC cells to TRAIL-driven cell death mainly by increasing the expression of DR5 and to a lesser extent DR4. One such compound is apigenin, a flavonoid widely distributed in many fruits and vegetables. Apigenin increases DR5 expression and synergistically acts with TRAIL in inducing DLD-1 cell death [45]. DR5 up-regulation also accounts for the TRAIL-sensitizing effect of both baicalein and isoliquiritigenin in SW480 and HT-29 cells, respectively [46,47]. Kaempferol, a flavonoid isolated from many plant sources (e.g., tea, broccoli, propolis, grapefruit), also shows TRAIL sensitizing effect in SW480 cells. Kaempferol up-regulates the expression of both DR4 and DR5 but knock-down of DR5 and not DR4 by siRNA effectively abrogates apoptosis induced by the combined treatment of this compound with TRAIL [48]. DR modulation partly underlies the TRAIL sensitizing effect of quercetin and wogonin [49,50]. Quercetin, a flavonoid found in vegetables, fruits and tea, aggregates DRs into lipid rafts thus facilitating the DISC formation without increasing DR expression at the cell surface [49]. Wogonin, an active ingredient of Chinese herb medicine *Scutellaria baicalensis*, up-regulates DR5 and decreases c-FLIP expression in HT-29 cells [50]. The TRAIL-sensitizing properties of other tested polyphenols are associated with modulation of different intracellular pro- and anti-apoptotic proteins. For example, cycloartenyl ferulate, a phenolic compound present in γ-oryzanol (a component of the rice bran oil), up-regulates DRs and increases caspase-8 activation as well as Bid cleavage leading to mitochondrial pathway activation in both SW480 and SW620 cells [51]. In the same cells, the flavonolignan silibinin used in combination with TRAIL synergistically induces cell death through both a p53-independent DR5 up-regulation and down-regulation of Mcl-1 and XIAP [52]. Another polyphenol recently reported to have TRAIL sensitizing properties in CRC cells is gossypol. Gossypol is derived from cottonseed oil and commonly designated as a BH3 mimetic due to its ability to bind the BH3 binding pockets of Bcl-2 and Bcl-xL thus inhibiting their function [78]. Beside the down-regulation of anti-apoptotic proteins involved in TRAIL resistance (e.g., Bcl-2, Bcl-xL, survivin), this molecule potentiates TRAIL-driven apoptosis in HCT-116 cells by increasing DR5 expression through the ROS-mediated induction of CHOP [53]. In the same cells, the ROS-CHOP-mediated up-regulation of DR4 and DR5 is needed for TRAIL sensitization by cardamonin. The potentiation of TRAIL-induced apoptosis by this chalcone also correlates with DcR1 down-regulation and modulation of both pro-apoptotic (*i.e*., Bax) and pro-survival (e.g., Bcl-2, c-IAP1) intracellular proteins [54].

#### 3.2.2. Terpenoids

Terpenoids are one of the largest families of natural products. Terpenoids contained in many plants, and in particular the subclasses of monoterpenes, diterpenes and tetraterpenes (including carotenoids), play a role in traditional herbal medicine and are currently under investigation for their antitumor activities [79,80]. Zerumbone, a sesquiterpene isolated from the rhizome of a tropical ginger, shows anticancer activity against different tumor cell types but very little or no cytotoxic effect on normal human endothelial cells and dermal fibroblasts [81]. In HCT-116 cells, which possess a functional p53, treatment with zerumbone generates ROS and subsequently activates the MAPK ERK1/2 leading to DR4/DR5 induction and TRAIL sensitization [55]. Interestingly, zerumbone-mediated DR up-regulation as well as the increased TRAIL-induced apoptosis is abolished by ROS scavenging compounds thus suggesting a key role of free radicals in these processes. Additionally, zerumbone markedly down-regulates the expression of c-FLIP without affecting other anti-apoptotic proteins (*i.e*., Bcl-2, Bcl-xL, XIAP, survivin) [55]. However, zerumbone shows no sensitizing effect on HT-29 cells that express mutant p53 [55]. An increased susceptibility to TRAIL-induced apoptosis is observed in both HCT-116 and HT-29 cells when treated with nimbolide, a tetranortriterpenoid isolated from the leaves and flowers of the tree *Azadirachta indica* [56]. Several mechanisms are proposed for this effect. First, nimbolide up-regulates the expression of DRs through ROS production and MAPK pathway activation in a p53-independent fashion. This up-regulation is due to the increase of both transcription and protein stability and is critical for the TRAIL sensitization by this compound. Indeed, knock-down of DRs, and in particular of DR5, significantly hampers the TRAIL-sensitizing effect of nimbolide. Second, nimbolide up-regulates Bax and potentiates the intrinsic pathway of apoptosis. Finally, nimbolide down-regulates the expression of critical anti-apoptotic proteins (*i.e*., c-FLIP, Bcl-2, Bcl-xL, c-IAP1, c-IAP2, survivin and XIAP) [56]. TRAIL-sensitizing properties are also reported for halocynthiaxanthin, a dietary carotenoid contained in oysters and sea squirts, in DLD-1 and HT-29 cells. This effect is associated with DR5 up-regulation further confirming the key role of this DR in the TRAIL apoptotic pathway [57].

#### 3.2.3. Other Natural Products

TRAIL-sensitizing effects in CRC cells are also reported for other natural products not belonging either to polyphenols or terpenoids, such as vitamin-related compounds. Among these compounds γ-Tocotrienol (γ-T3), an unsaturated tocopherol found in palm oil, rice bran, barley, and wheat germ, enhances TRAIL-induced apoptosis in HCT-116 cells through ERK1/2-driven ROS-dependent DR4/DR5 up-regulation [58]. Despite p53 not being activated by γ-T3, it is required in the DR up-regulation process as evidenced by the failure of γ-T3 to induce DRs both in p53 knockout HCT-116 cells and in the p53 mutated HT-29 cells [58]. A different mechanism, independent of the modulation of CHOP, p53, Bax and mitogen-activated protein kinases (MAPK), underlies both DR4 and DR5 up-regulation in CRC cells by garcinol, a compound derived from the dried rind of the fruit *Garcinia indica*. Moreover, garcinol diminishes expression of c-FLIP, Bcl-2, survivin and XIAP [59]. The down-regulation of anti-apoptotic proteins also account for the sensitization of CRC cells to TRAIL by combretastatin A-4 and the plant stress hormone methyl jasmonate [60,61]. Combined treatment of HT-29 cells with TRAIL and the polyunsaturated fatty acid docosahexaenoic acid (DHA) markedly increases cell death compared to the agents used alone [62]. On the molecular level, DHA dose-dependently induces ROS production, enhances caspase-8 activation and the formation of tBid [62]. HT-29 cells are sensitized to TRAIL-induced cell death also when exposed to diosgenin, a steroid saponin present in fenugreek (*Trigonella foenum graecum*) and other plants, through the increase of DR5 expression [63]. Zhang and colleagues demonstrated that the combination of TRAIL and the vitamin A-related compound all-*trans*-retinyl acetate (RAc) restores apoptosis in APC-deficient premalignant cells *in vivo* without affecting normal cells [64]. A similar effect was observed *in vitro* as RAc sensitizes human colon epithelial cells (NCM356) with altered APC function to TRAIL. Specifically, in these cells, RAc-mediated TRAIL sensitization is associated with DR up-regulation and suppression of both DcR1 and DcR2 thus leading to enhanced caspase-8 activation [64].

### 3.3. Other TRAIL-Sensitizing Agents

Other TRAIL-sensitizing molecules discussed in this article include proteasome inhibitors, ER stress inducers and molecules controlling p53, AMP-activated protein kinase, protein kinase C and the molecular chaperone Hsp90.

#### 3.3.1. Proteasome Inhibitors

Inhibition of the proteasome, a multicatalytic enzyme complex that recognizes and degrades poly-ubiquitinated proteins, has been proposed as a possible therapeutic strategy to manage human malignancies [82]. In this context, the proteasome inhibitor MG132 cooperates with TRAIL in inducing HCT-116 apoptosis through DR5 up-regulation [65]. Another proteasome inhibitor, PS-341, synergizes with TRAIL to enhance HCT-116 and HC4 cell death through DR up-regulation and the following activation of both extrinsic and intrinsic apoptotic pathways [66].

#### 3.3.2. ER Stress Inducers

Disruption of ER homeostasis due to various stress conditions leads to accumulation and aggregation of unfolded and/or misfolded proteins in the lumen of this organelle and to activation of an ER stress response termed unfolded protein response [83]. A large body of evidence indicates that ER stress inducing agents exert antitumor properties [84]. 15-Deoxy-delta (12,14)-prostaglandin J2 (15dPGJ2), a known ER stress inducer, increases DR5 expression thus sensitizing HCT-116 cells to TRAIL-induced apoptosis. DR5 up-regulation by 15dPGJ(2) is independent of PPAR-γ and p53 but relies on ROS-mediated CHOP induction [67]. Likewise, ROS and CHOP are critical for dibenzylideneacetone (DBA) to sensitize HCT-116 to TRAIL through DR4 and DR5 induction and down-regulation of anti-apoptotic proteins [68]. Moreover, DcR2 but not DcR1 is decreased by DBA probably contributing to TRAIL sensitization [68]. ER stress is also one of the mechanisms by which 2-Methoxy-5-Amino-N-Hydroxybenzamide (termed 2–14), a derivative of mesalamine, restores the susceptibility of CRC cells to TRAIL [69]. We have also shown that 2–14 inhibits CRC cell growth both *in vitro* and *in vivo* without affecting the proliferation of normal colonic cells, intraepithelial lymphocytes and fibroblasts [85]. The 2–14-mediated TRAIL sensitization is seen in the p53-mutant DLD-1 and HT-29 cells and this phenomenon is in part dependent on ERK1/2-driven CHOP-mediated DR5 up-regulation [69]. Additionally, 2–14 promotes the proteasome-mediated degradation of survivin, a major hurdle to TRAIL signaling in these CRC cell lines [69]. Both CHOP-mediated DR5 increase and survivin down-regulation are also the major mechanisms by which dipyridamole, a thromboxane synthase inhibitor widely used as an antiplatelet, sensitizes SW480 cells to TRAIL [70].

#### 3.3.3. Molecules Controlling p53, AMP-Activated Protein Kinase, Protein Kinase C and the Molecular Chaperone Hsp90

p53 activation can contribute to sensitize tumor cells to TRAIL-induced apoptosis [86]. Nutlin-3, a cis-imidazoline derivative, interferes with Mdm2-mediated p53 degradation with the downstream effect of promoting p53-dependent up-regulation of DR5 and enhancing HCT-116 susceptibility to TRAIL. The pro-apoptotic effect of nutlin-3 is also mediated by Bcl-2 and XIAP down-regulation [71]. A p53-dependent TRAIL sensitization is also reported in HCT-116 cells treated with 5-Aminoimidazole-4-carboxamide riboside (AICAR), an activator of AMP-activated protein kinase [72]. Co-treatment of these cells with AICAR and TRAIL reduces Bcl-2 expression and increases Bid cleavage thus potentiating the intrinsic mitochondrial pathway [72]. Furthermore, rottlerin and 17-allylamino-17-demethoxygeldanamycin (17-AAG), known to inhibit the protein kinase C and the molecular chaperone Hsp90 respectively, are effective in overcoming TRAIL resistance in HT-29 and RKO cells. This is achieved via direct effects at the level of the mitochondria and by reducing the expression of IAPs, and in particular XIAP [73,74].

## 4. Conclusions

Accumulating evidence indicates TRAIL is a promising anti-cancer agent due to its ability to selectively induce apoptosis in transformed cells. However, cancer cells including CRC cells often develop resistance to TRAIL-induced apoptosis and this issue has strongly limited the use of TRAIL-based therapies. Chemotherapeutic drugs are reported to overcome resistance of CRC cells to TRAIL but unfortunately their wide use is limited by the risk of side-effects. Therefore, the validation of effective TRAIL-sensitizing agents which exhibit diminished toxicity is worth pursuing. Several natural and synthetic compounds, which revert various cellular defects contributing to TRAIL resistance, have already been tested with success in CRC cell systems. An important issue relates to the choice of which compound should be used. Since resistance of CRC cells to TRAIL can rely on the presence of multiple defects, we can speculate that compounds that target more pathways simultaneously may have higher potential for anticancer therapy. It would be also useful to better characterize such defects in single patients in order to personalize TRAIL-based therapy. Another concern deals with compounds which increase p53 expression. Indeed, up-regulation of DcRs by p53 may result in a significantly delay of TRAIL-induced killing thus limiting the use of such agents in CRC tumors bearing functional p53. Additionally, hypoxic conditions, easily found in solid tumors, may render CRC cells resistant to some therapeutic agents and attenuate TRAIL-induced cell death. All these drawbacks should be taken into account before designing combined therapies involving TRAIL. Further experimentation is however needed to confirm the *in vitro* data in pre-clinical models of CRC and ascertain the profile of safety and tolerability of such compounds.

## Figures and Tables

**Table 1:** Mechanism(s) of action of TRAIL-sensitizing agents in CRC cells.

Therapeutics	Cells	Mechanism(s)	Reference
***Chemotherapeutics***			
Cisplatin	HT-29, SW480	Caspase-8 activation, Bid cleavage	[35,36]
5-FU	HCT-116, HT-29	Caspase-8 activation, ↓c-FLIP, Bid cleavage	[35–38]
VP16	HCT-116, SW480	Caspase-8 activation	[39]
Doxorubicin	HT-29, SW480	Caspase-8 activation, Bid cleavage	[35,36]
DuP-697	HT-29	DR5 and DISC component redistribution in lipid rafts	[25]
CPT-11	HCT-116	↑DR4, ↑DR5	[40]
Fenretinide	HT-29, SW480	↑DR5, Bid cleavage	[41]
Lapatinib	HCT-116, SW480, SW620, DLD-1, HT-29	↑DR4, ↑DR5	[42]
Sunitinib	SW620	↓c-FLIP, ↓Mcl-1, ↓XIAP	[43]
Oxaliplatin	HT-29, V9P	↓Bcl-xL anti-apoptotic activity	[44]
***Natural products***			
Apigenin	DLD1	↑DR5	[45]
Baicalein	SW480	↑DR5	[46]
Isoliquiritigenin	HT-29	↑DR5	[47]
Kaempferol	SW480	↑DR5	[48]
Quercetin	HT-29, SW620, CACO-2	DR4 and DR5 redistribution in lipid rafts	[49]
Wogonin	HT-29	↑DR5, ↓c-FLIP	[50]
Cycloartenyl ferulate	SW480, SW620	↑DR4, ↑DR5, Bid cleavage, Bcl-2, Bax	[51]
Silibinin	SW480, SW620	↑DR5, ↓Mcl-1, ↓XIAP	[52]
Gossypol	HCT-116	↑DR5, ↓Bcl-2, ↓Bcl-xL, ↓survivin	[53]
Cardamonin	HCT-116	↑DR4, ↑DR5, ↓DcR1, ↑Bax, ↓Bcl-2, ↓c-IAP1	[54]
Zerumbone	HCT-116	↑DR4, ↑DR5, ↓c-FLIP	[55]
Nimbolide	HCT-116, HT-29	↑DR4, ↑DR5, ↓c-FLIP, ↑Bax, ↓Bcl-2, ↓Bcl-xL, ↓c-IAP1, ↓c-IAP2, ↓survivin, ↓XIAP	[56]
Halocynthiaxanthin	DLD1, HT-29	↑DR5	[57]
γ-T3	HCT-116	↑DR4, ↑DR5	[58]
Garcinol	HCT-116, HT-29	↑DR4, ↑DR5, Bid cleavage ↓c-FLIP, ↓Bcl-2, ↓XIAP, ↓survivin	[59]
Combretastatin A-4	HCT-116, SW620	↓c-FLIP, ↓Mcl-1	[60]
Methyl jasmonate	HCT-116, SW480	Bid cleavage, ↓survivin	[61]
DHA	HT-29	Caspase-8 activation, Bid cleavage	[62]
Diosgenin	HT-29	↑DR5	[63]
RAc	APC-deleted NCM356	↑DR4, ↑DR5, ↓DcR1, ↓DcR2	[64]
***Other agents***			
MG-132	HCT-116	↑DR5	[65]
PS-341	HCT-116, HC-4	↑DR4, ↑DR5, caspase-8 activation, Bid cleavage	[66]
15dPGJ_2_	HCT-116	↑DR5	[67]
DBA	HCT-116	↑DR4, ↑DR5, ↓DcR2, ↓Bcl-2, ↓XIAP, ↓survivin	[68]
2–14	DLD1, HT-29	↑DR5, ↓survivin	[69]
Dipyridamole	SW480	↑DR5, ↓survivin	[70]
Nutlin-3	HCT-116	↑DR5, ↓Bcl-2, ↓XIAP	[71]
AICAR	HCT-116	Bid cleavage, ↓Bcl-2	[72]
Rottlerin	HT-29, RKO	Cytochrome c and Smac/DIABLO release, ↓XIAP ↓c-IAP1	[73]
17-AAG	HT-29, RKO	Bid cleavage, ↓XIAP	[74]
